# Zwitterionic Tröger’s Base Microfiltration Membrane Prepared via Vapor-Induced Phase Separation with Improved Demulsification and Antifouling Performance

**DOI:** 10.3390/molecules29051001

**Published:** 2024-02-25

**Authors:** Meng Wang, Tingting Huang, Meng Shan, Mei Sun, Shasha Liu, Hai Tang

**Affiliations:** School of Chemical and Environmental Engineering, Anhui Polytechnic University, Wuhu 241000, China; wm18855372043@163.com (M.W.); 18895367471@163.com (T.H.); 15551732072@163.com (M.S.); sunmei17855461938@163.com (M.S.)

**Keywords:** zwitterionic polymer, Tröger’s base, antifouling, demulsification, microfiltration membrane

## Abstract

The fouling of separation membranes has consistently been a primary factor contributing to the decline in membrane performance. Enhancing the surface hydrophilicity of the membrane proves to be an effective strategy in mitigating membrane fouling in water treatment processes. Zwitterionic polymers (containing an equimolar number of homogeneously distributed anionic and cationic groups on the polymer chains) have been used extensively as one of the best antifouling materials for surface modification. The conventional application of zwitterionic compounds as surface modifiers is intricate and inefficient, adding complexity and length to the membrane preparation process, particularly on an industrial scale. To overcome these limitations, zwitterionic polymer, directly used as a main material, is an effective method. In this work, a novel zwitterionic polymer (TB)—zwitterionic Tröger’s base (ZTB)—was synthesized by quaternizing Tröger’s base (TB) with 1,3-propane sultone. The obtained ZTB is blended with TB to fabricate microfiltration (MF) membranes via the vapor-induced phase separation (VIPS) process, offering a strategic solution for separating emulsified oily wastewater. Atomic force microscopy (AFM), scanning electron microscopy (SEM), water contact angle, and zeta potential measurements were employed to characterize the surface of ZTB/TB blended membranes, assessing surface morphology, charge, and hydrophilic/hydrophobic properties. The impact of varying ZTB levels on membrane surface morphology, hydrophilicity, water flux, and rejection were investigated. The results showed that an increase in ZTB content improved hydrophilicity and surface roughness, consequently enhancing water permeability. Due to the attraction of water vapor, the enrichment of zwitterionic segments was enriched, and a stable hydration layer was formed on the membrane surface. The hydration layer formed by zwitterions endowed the membrane with good antifouling properties. The proposed mechanism elucidates the membrane’s proficiency in demulsification and the reduction in irreversible fouling through the synergistic regulation of surface charge and hydrophilicity, facilitated by electrostatic repulsion and the formation of a hydration layer. The ZTB/TB blended membranes demonstrated superior efficiency in oil–water separation, achieving a maximum flux of 1897.63 LMH bar^−1^ and an oil rejection rate as high as 99% in the oil–water emulsion separation process. This study reveals the migration behavior of the zwitterionic polymer in the membrane during the VIPS process. It enhances our comprehension of the antifouling mechanism of zwitterionic membranes and provides guidance for designing novel materials for antifouling membranes.

## 1. Introduction

In the past two decades, significant quantities of emulsified oily wastewater have been released from petrochemical, steel, and other industrial processes in China, which have caused severe environmental pollution in water ecosystems [1,2,3]. The conventional treatment technologies for emulsified oily wastewater include chemical demulsification, air flocculation, and biochemical treatment methods. Microfiltration (MF) membranes are commonly utilized for separating emulsified oily wastewater treatment due to their short separation time, low energy consumption, high efficiency, lack of additional chemicals, and convenient operation [4]. However, oil droplets frequently adhere to the hydrophobic membrane surface, exacerbating membrane fouling and increasing separation difficulty [5,6].

Several studies have shown that hydrophilic and charged MF membranes can effectively treat surfactant-stabilized emulsions (SSEs) [7,8,9,10,11,12]. Lin et al. [13] prepared a modified PEI electrospun fiber membrane, and the positive potential point generates electrostatic repulsion with the cationic surfactant molecules in the emulsion. The use of zwitterionic polymers reduces the adhesion of the surfactant to the membrane, resulting in decreased pollution and increased permeation flux. Zwitterionic polymers are also excellent materials for surface hydrophilization [14,15,16,17], and previous research has indicated that negatively charged zwitterionic-modified blended MF membranes show potential for treating SSE with excellent separation performance [8,18,19,20]. Maggay et al. [21] prepared zwitterionic PVDF membranes using a novel polymer made of styrene units and zwitterionic 4-vinylpyridine. The material exhibited exceptional anti-biofouling properties against various biofoulants. Zhu et al. [22] prepared a zwitterionic PTMAO-grafted PVDF membrane using the vapor-induced phase separation (VIPS) method. The results revealed that the presence of zwitterionic segments on the membrane surface attracted water vapor, resulting in a closely bound hydration layer on the membrane surface. This had led to strong oil repellency in water [23].

Recently, it has been shown that VIPS offers significant advantages in regulating membrane morphologies. This is due to the slower kinetics of the gaseous phase and non-solvency during the phase separation of the membrane [24,25]. This provides control over the phase separation process by adjusting the polymer concentration, vapor exposure time, and temperature [26,27,28]. Poly(vinylidene fluoride) (PVDF) has been widely used to prepare MFs for oil/water emulsions [29,30] due to its ability to achieve high porosity (>70%). For example, Chen et al. [31] reported a novel approach for regulating the pore structure of MF membranes via lowering the solution temperature. The results showed that elevating the temperature facilitated the formation of cell-like pores, resulting in an ultrahigh flux of 3028 LMH bar^−1^. Nevertheless, the use of VIPS for MF membranes still presents practical challenges related to the regulation of pore structure, achieving optimal demulsification efficiency, and improving antifouling performance [27].

In our previous study, we regulated the structure and performance of a UF membrane derived from Tröger’s base (TB) by blending different contents of zwitterionic TB (ZTB). The results suggested that the zwitterionic TB polymer enhanced the permeability and antifouling performance of the UF membrane [32]. This work investigated the potential applications of a TB polymer-based MF membrane via VIPS. This work explored the effects of the molar ratio of TB and ZTB, temperature, and vapor exposure time on the membrane’s morphology, including porosity and pore size. The surface charge and hydrophilicity of the membrane surface were easily controlled. The hydrophilicity, surface roughness, and morphology of the membrane surface and cross-section were studied by using water contact angle (WCA), atomic force microscopy (AFM), and scanning electron microscopy (SEM). The performance of surfactant-stabilized emulsions, in terms of membrane permeability, rejection, and antifouling performance, were also analyzed. Additionally, the demulsification mechanism was examined. This work aims to enhance the demulsification and antifouling performance of MF membranes by blending TB and ZTB to fabricated zwitterionic MF membranes.

## 2. Results and Discussion

### 2.1. MF Membrane Morphology

**Figure 1:**
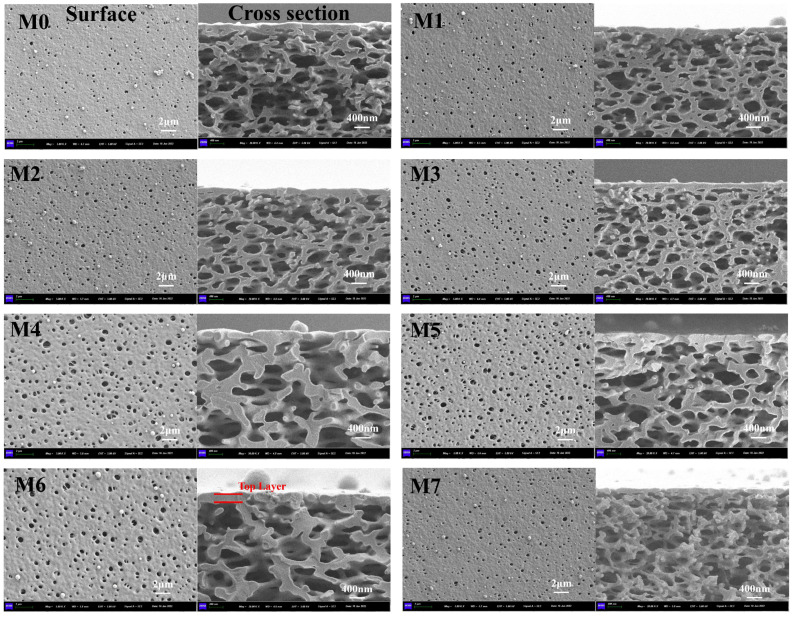
Surface and cross-sectional SEM images of membranes.

In the VIPS method of preparation, it is notable that when the cast membrane is exposed to controlled humidity for a specific duration, the upper surface undergoes alteration due to the absorption of water vapor droplets, while the integrity of the majority of the polymeric cast film remains unaffected. Following precipitation in a coagulation nonsolvent bath, the changes observed extend throughout the entirety of the resulting membrane. Thus, the precise control of the conditions during the initial phase separation in the humid chamber impacts the formation pattern, surface pore size, and overall structure of the membrane. Water vapor droplets in a humid environment can leave distinct marks on soft membrane surfaces due to their high mobility and ability to deform the surface. The intensity of these marks can result in various morphologies depending on the specific conditions present. During VIPS membrane formation, due to the strong interactions between the solvent (NMP) and water vapor, when the water vapor enters the polymer solution, the surface layer of the membrane quickly precipitates. Either initial pores on the polymeric membrane or a coagulated surface could form. A thin layer of gel then forms, hindering the exchange of solvent and non-solvent, inhibiting the formation of macropores in the membrane and causing rough and uneven sponge-like holes to appear on the surface [31,33,34,35]. Similar results have been obtained in the preparation of high-strength PVDF porous membranes with a cellular structure via VIPS. The findings demonstrate that the membrane’s pore size exhibits variability in response to exposure duration, temperature fluctuations, and additional environmental parameters [36]. The surface and cross-sectional SEM images of the M0–M7 membranes were observed and are shown in Figure 1, which shows that the cross-section of the final MF membranes has a sponge-like structure. The surface porosity (*P*_s_), average surface pore size (*r*_s_), maximum surface pore size (*r*_max_), and top-layer thickness (*T*) of the membranes are summarized in Table 1. The *P*_s and_ *r*_s_ of M0 (pristine TB) are 1.2% and 0.178 μm, respectively.

**Figure 2:**
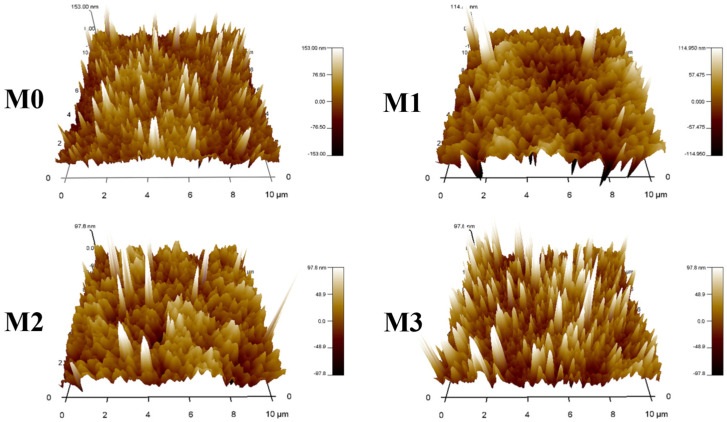
AFM images of membrane surfaces.

It was observed that the zwitterionic polymers significantly influenced the surface porosity. The surface porosity of the M0 membrane was 1.2 ± 0.2%, while that of the blended MF membranes gradually increased to 5.1 ± 0.1%, which was 1.4–4.4 times that of M0. The average surface pore size of M0 was 0.178 ± 0.002 μm, and the pore size in the blended MF membrane increased from 0.203 ± 0.001 μm to 0.247 ± 0.005 μm. This occurred because the phase separation rate of the blended membrane was slower and the hydrophilicity of the MF membrane gradually increased. As a result, more water vapor was required to permeate into the membrane [32,37], and the porosity increased. The different interactions of the hydrophilic ZTB and the hydrophobic Tröger’s base polymer during the phase transformation led to a nanoscale microphase separation [38] and gave the MF membranes a relatively uniform size [39]. Furthermore, Figure 2 shows AFM images of the M0–M3 MF membrane surface, and Table 2 shows the average surface roughness (*Ra*) and root mean square roughness (*Rq*) of the membrane surface. *Ra* and *Rq* of the blended membranes increased with increasing numbers of ZTB polymers. *Ra* increased from 21.79 nm for M0 to 24.24 nm for M3, and *Rq* increased from 29.57 nm for M0 to 45.93 nm for M3, which was 1.6 times that of M0. This indicates that the surface roughness of the MF membrane gradually increased as the ZTB content increased. Previous studies have also shown that delayed phase separation allows sufficient time for the rich/poor polymer to grow before solidifying, resulting in a rough membrane surface [40] that improves membrane permeability [7].

Exposure time to solvent vapor has an important influence on membrane morphology [35], which was investigated by comparing M2, M4, and M5 membranes. As the *t*_e_ increased from 5 min to 10 min (M2), the amount of condensed water vapor on the membrane surface increased, which decreased the mass transfer resistance (concentration gradient) and the phase separation rate compared with the M4 membrane. Then, the pore size became smaller, and the pore size distribution was narrow [41]. When *t*_e_ continued to increase to 15 min (M5), the pores connected to each other to form larger pores. The slow penetration of the non-solvent may have caused this, with delayed phase separation controlling the lean phase of the polymer and contributing to the formation of highly porous membranes [9]. When exposed to humid air, the polymer membrane experienced water vapor condensation on its surface, resulting in slight phase separation. Subsequently, upon immersion in the coagulation bath, the top layer solidified rapidly, inhibiting the exchange of solvent and non-solvent, resulting in the disappearance of the dense top layer [25,37]. When *t*_e_ = 5 min (M4), there were insufficient condensed water droplets to form a gel layer, but the absorbed water served as the foundation for pore formation, thus promoting phase separation in the coagulation bath and the formation of macropores [42,43,44]. Prolonged exposure facilitated the crystallization process, which led to the development of a porous skin and particle morphology. This, in turn, enhanced the surface hydrophobicity [28].

M2, M6, and M7 MF membranes were compared to explore the effects of exposure temperature. A higher temperature shortened the polymer’s gel time, preventing water vapor from infiltrating the solution [45]. At 30 °C (M6), the structure of finger and sponge pores underwent a phase transition, resulting in the formation of a thick surface layer and large cross-section pores due to the movement rate of the water molecules. Nevertheless, the rate of movement of the water vapor molecules accelerated when the temperature reached 50 °C. The formation of a gel layer on the membrane surface resulted in a decrease in the rate of phase separation. This, in turn, led to the formation of a thin epidermal layer and a reduction in the number of cross-section pores. At 80 °C (M7), the system’s thermodynamic instability was worsened by higher temperatures, causing the phase transformation to accelerate more than the delayed phase separation effect of the ZTB polymer. The growth time of the polymer lean phase decreased, and the interaction time of the polymer chain also decreased. The structure of the MF membrane transitioned from a bicontinuous sponge-like structure to a sponge structure over time.

### 2.2. Hydrophilicity of MF Membrane

**Figure 3:**
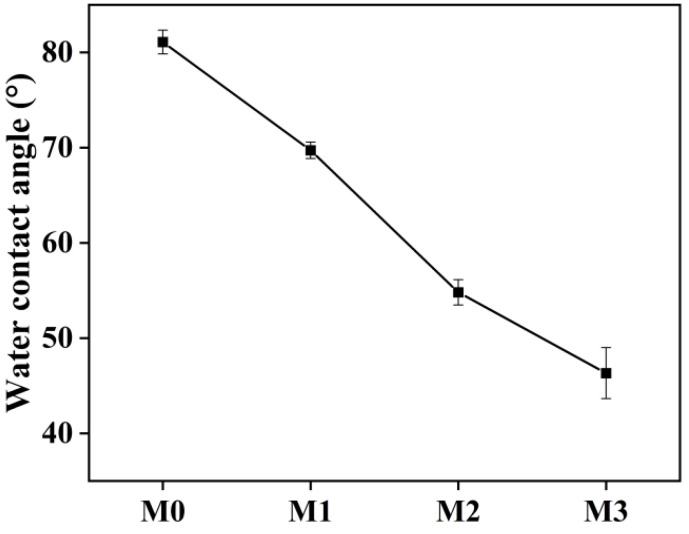
WCAs of membranes with different ZTB contents.

Previous studies have demonstrated that the hydrophilization and charge of the membrane surface can enhance the demulsification and antifouling performance of oil-in-water emulsions. This prevents foulants from adhering to the membrane due to significant steric hindrance [10,46]. Therefore, the wettability of membranes used for separating oil-in-water emulsions is a critical property [47]. The WCAs of membranes with different ZTB polymer contents are shown in Figure 3. The results show that the static WCA of the TB membrane (M0) was 81.1°, while that of membranes M1–M3 decreased to 69.72°, 54.81°, and 46.32°, respectively, upon increasing the ZTB polymer content from 1.0 wt% to 3.0 wt%. Similarly, −∆*G*_ML_ (Table 2) increased from 83.0 mJ m^−2^ for M0 to 118.1 mJ m^−2^ for M3. Therefore, adding the ZTB polymer significantly improved the hydrophilicity of the MF membrane [32].

### 2.3. Zeta Potential of Oily Wastewater and MF Membrane

**Figure 4:**
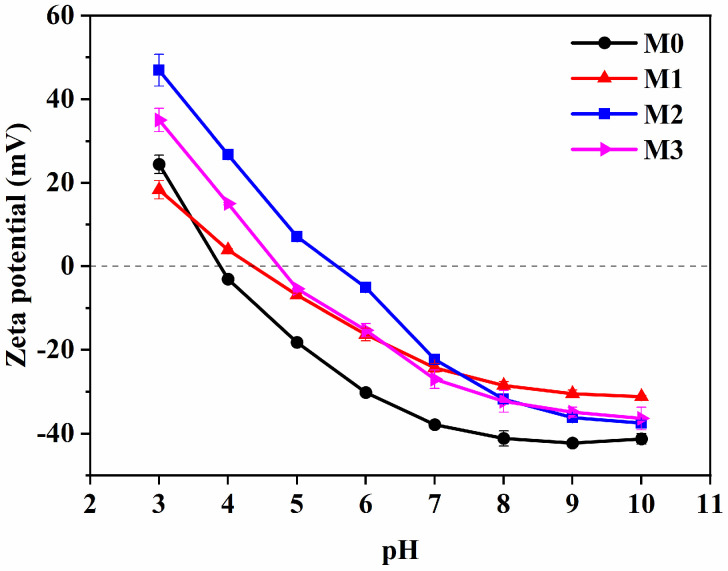
Zeta potential of M0–M3 MF membranes at pH 3–7.

SDS is an anionic surfactant that exhibits a negative charge in PBS solution (pH 7.4), resulting in a zeta potential of −55.5 mV when added to emulsified oily wastewater. The zeta potential values of the M0–M3 MF membranes in the pH range of 3.0–10.0 are shown in Figure 4. The hydrophilic MF membrane with zwitterionic properties displayed varying surface zeta potential values. The M0 membrane surface exhibited a negative charge within the pH range of 4.0–10.0 with a zeta potential of −3.1 mV to −41.3 mV because of the protonation of the tertiary amine groups of TB under acidic conditions, which increased the positive charge density on the membrane surface [48]. After the addition of the ZTB polymer, the isoelectric point of the membrane gradually tended to electrical neutrality. –SO_3_H remained uncharged, and the quaternary amine group showed a positive charge (–C–N^+^) under acidic conditions. The –SO_3_H group was negatively charged, and the quaternary amine group was neutral under alkaline conditions. The negatively charged membrane surface indicates that the introduction of a zwitterionic polymer weakened the electronegativity, in accordance with our previous report [14]. We also found that the zeta potential of the M3 membrane was slightly lower than that of the M2 membrane but still higher than that of the M0 membrane because the reaction consumed the tertiary amine groups on the membrane surface.

### 2.4. Penetration and Rejection Performance

**Figure 5:**
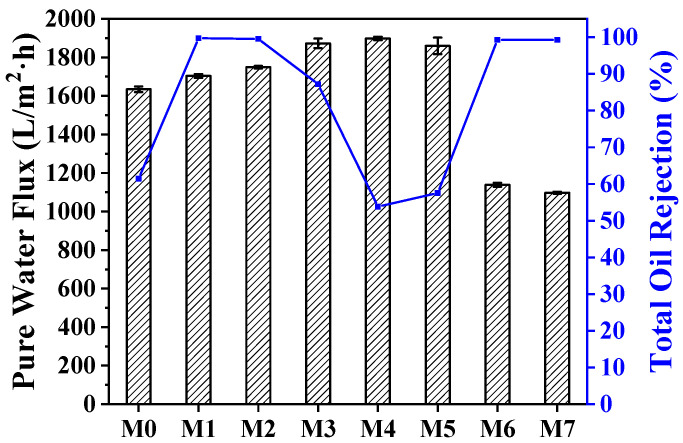
Flux and rejection rates of membranes fabricated using different parameters.

Three cycles of membrane performance measurement experiments were conducted using a cross-flow filtration system to assess the impact of ZTB addition, exposure time, and temperature on the permeability and rejection performance of the MF membranes. Figure 5 displays the flux and rejection rates of membranes fabricated using different parameters. The water flux of the M0 MF membrane reached 1634.34 LMH bar^−1^, but after adding the ZTB polymer, the water flux gradually increased from 1703.44 LMH bar^−1^ for M1 to 1872.97 LMH bar^−1^ for M3. The membrane’s surface porosity and pore size gradually increased, indicating that the ZTB polymer content could modulate the membrane’s microstructure, which, in turn, altered its permeability. The rejection rate of M0 for SSE was 61.47%, while that of M1–M3 was 99.67%, 99.53%, and 87.19%, respectively. The blended MF membranes exhibited significantly improved rejection rates for emulsified oil wastewater while maintaining high penetration. Additionally, they effectively separated emulsified oil droplets from solutions containing surfactants.

This study investigated the impact of exposure time and temperature on membrane performance. The flux of the UF membrane (*t*_e_ = 0 min) was significantly lower than that of the MF membrane. The fluxes of M4 (5 min) and M5 (15 min) were slightly higher than that of M2 (10 min), with rejection rates of emulsified oil droplets of 53.82%, 99.53%, and 57.55%, respectively. Different exposure times led to different membrane structures, and the pore size distribution ranges of the M4 and M5 blended MF membranes were larger than that of the M2, but the emulsified oil droplet particle size range was between 0.171 μm and 0.266 μm. Therefore, the oil droplets were able to pass through the membrane pores easily, resulting in a significant reduction in the rejection rate of emulsified oil. The fluxes of M2, M6, and M7 were 1138.76 LMH bar^−1^, 1748.61 LMH bar^−1^, and 1097.49 LMH bar^−1^, respectively. The SSE rejection rates of all the membranes were above 99%. The fluxes of the M6 and M7 membranes were lower than that of the M2, which might be due to the higher membrane surface thickness (1.311 μm) and lower surface porosity. The water flow through the membrane was restricted due to the large hydraulic resistance.

### 2.5. Antifouling Performance of Membranes

**Figure 6:**
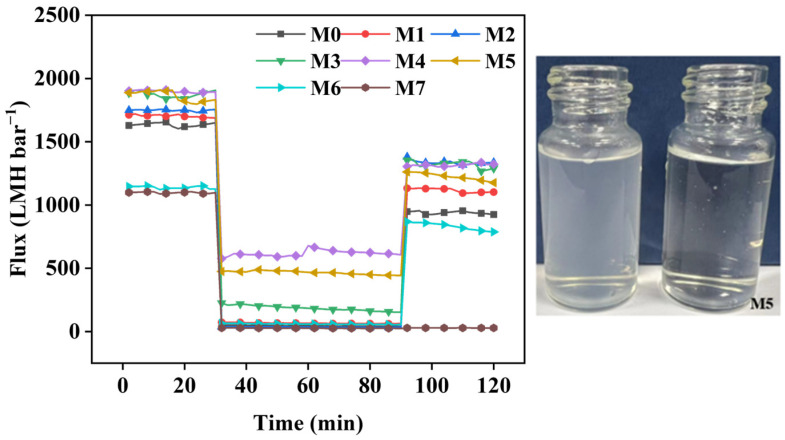
Pure water flux of the membrane using emulsified oil.

**Figure 7:**
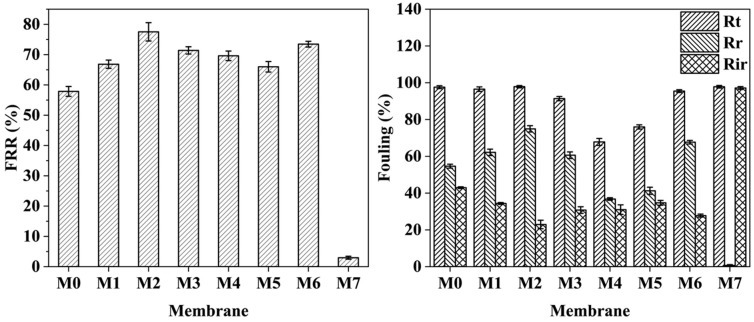
Antifouling performance of membranes fabricated using different parameters.

The effectiveness of membranes in separating oil-in-water emulsions depends critically on their antifouling performance [49]. FRR, *R*_t_, *R*_r_, and *R*_ir_ are important indicators for judging the antifouling performance of a membrane. Figure 6 and Figure 7 illustrate the pure water flux and antifouling performance of membranes when using emulsified oil, respectively. The FRR value of M0 was 57.5%, while the FRR values of the M1–M3 MF membranes exceeded that of M0. The FRR value of the M2 membrane was the highest (76.4%). The *R*_ir_ of the M1–M3 MF membranes slightly decreased upon increasing the ZTB content, and the *R*_ir_ of M2 reached the lowest value (23.6%). This demonstrates that adding the ZTB polymer improved the hydrophilicity of the membrane surface. Therefore, the FRR of the membrane rose significantly, and the antifouling performance was enhanced after washing with a NaOH solution (0.05 M) and distilled water. The FRR value of the M3 MF membrane decreased because it had the largest ZTB content, which may be due to the large increase in surface porosity and having the largest pore diameter on the membrane surface. During the cleaning process, it was difficult to remove blockages from the membrane surface due to the accumulation of large oil droplets that passed through the membrane pores. The FRR values of the M4 and M5 MF membranes were 69.32% and 65.86%, and their *R*_ir_ values were 30.68% and 34.14%, respectively. This confirmed that the antifouling performance of the membrane was directly related to the VIPS exposure time. At *t*_e_ = 0 min, the pore size of the membrane was smaller than the emulsified oil’s particle size, resulting in the emulsified oil forming a filter cake layer on the membrane surface. This caused a sharp decrease in the membrane flux, an increase in irreversible fouling, and a decrease in reversible fouling. The M2 MF membrane exhibited mainly reversible fouling because of its small average pore size.

The antifouling performance of the M2, M6, and M7 MF membranes was analyzed to investigate the influence of exposure temperature. This study aimed to determine how exposure temperature affects the performance of the membranes. The FRR value of M6 was 73.0%, which was slightly lower than that of M2. The FRR value of M7 decreased to 2.6% due to a decrease in the resistance of water vapor diffusion into the film-forming solution when the temperature rose to 80 °C. The membranes that were prepared at higher temperatures formed cellular structures, while the polymers became denser and finer [33,40,50]. The oil particles in the wastewater were transported by external pressure and penetrated the support sublayer of the membrane through pores on its surface. This internal structure of the polymer was reached by the oil particles. They partially obstructed the spaces between polymer chains. Therefore, when cleaning the membrane, only oil particles on the surface layer of the membrane could be removed, while those between the deep layers of the polymer chains could not be removed. This ultimately made it difficult to continuously filter water, thus obtaining an extremely low FRR value and extremely high *R*_r_ value.

### 2.6. Possible Antifouling and Demulsification Mechanism

**Figure 8:**
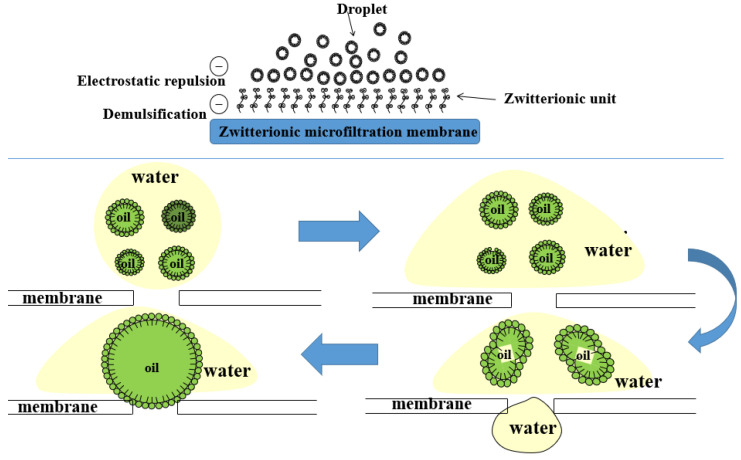
Demulsification mechanism of zwitterionic membranes.

The previous literature on the permeate flux and oil rejection of oil–water separation membranes is summarized in Table 3. Generally, the pore size, surface wettability, surface charge, and membrane structure of membrane materials play a crucial role in selective separation and demulsification using membranes. The blended MF membranes containing ZTB demonstrated highly effective performance in demulsifying emulsified oily wastewater. The possible demulsification mechanism is suggested in Figure 8. The hydrophilic and charged membrane surface resulted in size screening and wetting coalescence effects, which contributed to the high separation and anti-fouling performance of the emulsified oily wastewater [4]. The zwitterionic polymer combined with water molecules to form a hydration layer by solvating ionic groups. As a result, the aqueous phase in the emulsified oil wastewater wets and spreads preferentially on the membrane’s surface and pores. The hydration layer was established by the permeation of the interior, which enhanced the membrane’s antifouling performance. Under pressure, the aqueous phase penetrated the membrane pores, while collisions and squeezing between oil droplets deformed them simultaneously [51]. Moreover, the zwitterionic polymer endowed the membranes with a surface charge that destabilized emulsified oil and prevented oil from adhering to the membrane surface due to electrostatic repulsion. The molecules of the emulsifier at the membrane interface were partially removed or rearranged due to electrostatic repulsion. This facilitated the coalescence of the oil droplets. The large oil droplets in the emulsion underwent demulsification due to a gradual change in particle size and formed free oil droplets.

## 3. Materials and Methods

### 3.1. Materials and Chemicals

Dimethoxymethane (98.0%), 3,3′-dimethylbiphenyl-4,4′-diamine (98.0%), *o*-xylidine (98.0%), N-methyl-2-pyrrolidone (NMP, >99.0%), trifluoroacetic acid (TFA, 99.0%), and 1,3-propane sulfonic acid lactone (99%) were obtained from Aladdin Industrial, Co. Methanol (CH_3_OH, >99.7%), ammonia (NH_4_OH, 25–28%), chloroform (CHCl_3_, >99.0%), diethyl ether (C_4_H_10_O, >99.5%), sodium dodecyl sulfate (SDS), and sodium hydroxide (NaOH) were purchased from Chinese Medicine Co. (Shanghai, China) The chemicals were used in their original state without additional purification. The relative humidity (RH) during the VIPS process was achieved by adding water vapor to the membrane formation chamber.

### 3.2. Preparation of MF Membranes

**Figure 9:**
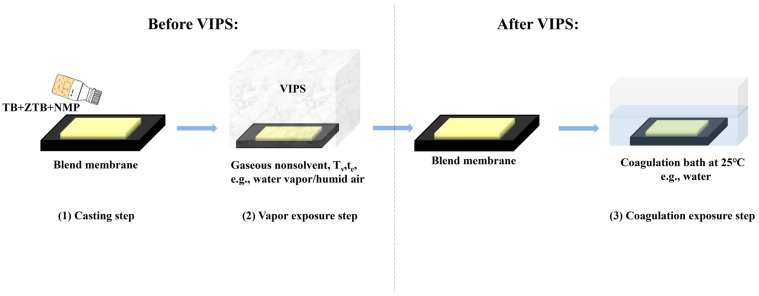
Illustration of the VIPS process used to prepare MF membranes.

The synthesis and characterization of ZTB is described in our previous work [32]. All MF membranes were prepared using the VIPS method. A typical preparation process is illustrated in Figure 9. The compositions and preparation conditions of the casting solution was listed in Table 4. Briefly, TB and ZTB polymers were added in a fixed molar ratio to 8.2 g NMP and stirred continuously at 25 °C for 12 h until dissolved completely. Then, they were defoamed in a vacuum-drying oven at 60 °C for 3 h to form a uniform casting solution. The obtained solution was poured onto a clean glass plate, and a scraper was used to generate a film with a thickness of 200 μm at a speed of 1.5 m min^−1^. After exposure to humid air for 10 s, the film was transferred to a constant temperature and humidity chamber with a relative humidity (RH) of 90%. Then, it was placed into deionized water at 25 °C until completely exfoliated from the glass plate. Subsequently, the membrane was kept in deionized water for 48 h and it was replaced regularly to completely remove any NMP solvent remaining on the membrane.

### 3.3. Membrane Characterization

The hydrophilicity of the membrane surface was assessed according to its water contact angle (WCA; OSA60, Beijing Eastern-Dataphy Instruments Co.). At ambient temperature, 5 μL water droplets were dropped onto the membrane surface. After 10 s, images of the droplets were taken with a camera, and the WCA was calculated using imaging software. The average value was calculated by measuring five different positions on the membrane, and the membrane’s liquid interface’s free energy −∆*G_ML_* (mJ m^−2^) was calculated using the modified Young–Dupré equation to measure its surface wettability [11,58], as shown in Formula (1).

(1) $$\Delta G_{ML}=\gamma_{L}(1+\frac{\cos\theta }{1+SAD})$$

where *γ*_L_ is the surface tension of water (72.8 mJ m^−2^, 20 °C); *θ* is the average WCA; and 1 + *SAD* (a roughness area parameter) is the ratio of the actual area to the geometric area of the membrane surface.

Atomic force microscopy (AFM; Bruker Dimension Edge) was used to measure the surface roughness of the membrane. The average roughness (*Ra*) and root mean square roughness (*Rq*) of the membrane were measured from the AFM images of three different positions on the membrane surface using Gwyddion 2.48 software. The morphology and surface and cross-sectional structures of the membranes were observed by SEM (S-4800). Before observations, all membrane samples were sputtered with gold. The membrane samples were frozen and made brittle using liquid nitrogen to obtain a flat membrane cross-sectional structure. The average pore size and porosity of the membrane surface were quantitatively calculated using ImageJ v1.48 software.

### 3.4. Simulated Stabilized Oil-in-Water Emulsions

**Figure 10:**
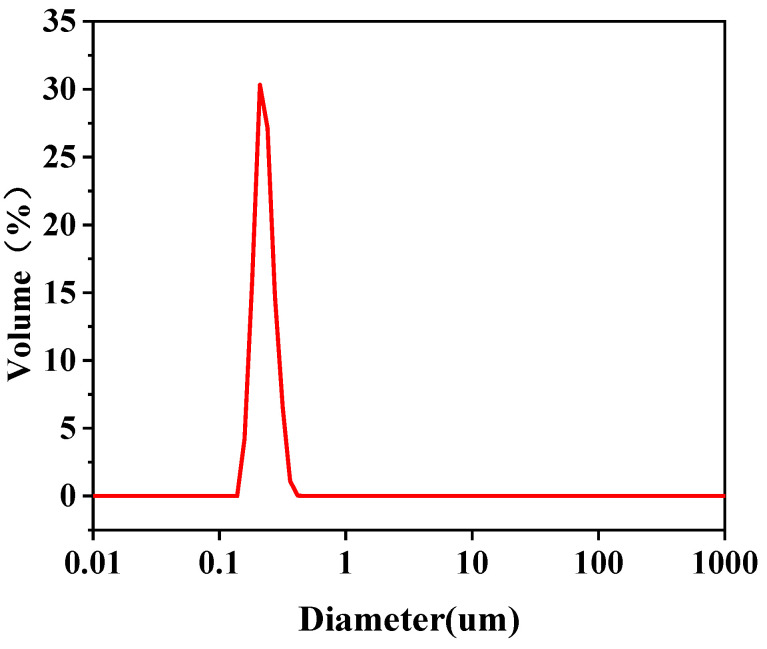
Particle size distribution of oil droplets in emulsions.

Cutting oil (0.05 g) and 0.05 g anionic surfactant sodium dodecyl sulfate (SDS) were added to a beaker and then ultrasonically mixed for 20 min. Then, the solution was stirred for 36 h at 500 rpm until a 0.05 g L^−1^ uniform yellow emulsion was obtained. To avoid suspension or separation of oil droplets during storage, the emulsions were configured 36 h before use. The size of the emulsified oil droplet was measured using a laser particle sizer (Master 2000). The results in Figure 10 and Table 5 show that the size distribution of the emulsified oil droplet was in the range of 0.171–0.266 μm, with a median average particle diameter *D*_50_ of 0.209 μm. This indicated that the particle size range of the prepared SSEW was narrow, and the overall oil droplet particle size was uniform.

### 3.5. Zeta Potential of the MF Membrane and Emulsified Oil

The zeta potential on the surface of the MF membrane was measured using a SurPASS solid surface zeta potential meter (Malvern Zetasizer Nano ZS90, Hefei, China). At ambient temperature, 1 mM KCl solution was employed as the electrolyte, and 5 mm HCl and NaOH solutions were used to adjust the pH in the range of 3–10. The test pressure was set to 30 kPa in terms of flowing current, and the two samples faced each other so that the slit spacing was controlled to 90–110 µm. Tests were repeated twice in the left and right directions. The zeta potential of emulsified oil wastewater was measured using a nanoparticle size analyzer (ZS-90, Malvern, UK). A certain content of samples was dispersed in deionized water (0.1 wt%), and ultrasonic oscillation was carried out for 20 min. The zeta potential was measured three times. All experiments were reported as the average values of three replicates.

### 3.6. Membrane Filtration and Antifouling Performance

The permeability, antifouling, and rejection performance of the MF membrane to the emulsified oil were measured using a cross-flow filtration device. The effective membrane area was 19 cm^2^. The MF membrane was pre-pressed with deionized water for 30 min at a pressure of 0.15 MPa to obtain a stable pure water flux. Subsequently, the pressure was reduced to 0.1 MPa, and filtration was continued for 30 min to obtain the initial pure water flux of the membrane. Then, emulsified oil wastewater was treated as the feed liquid for 1.0 h. After that, the membrane was washed with 0.05 M NaOH solution for 5 min, and then deionized water for 25 min to remove residual NaOH. The membrane was filtered again with deionized water for 30 min to test the pure water flux recovered. The membrane flux *J* (LMH bar^−1^) was calculated using Formula (2):

(2) $$J=\frac{m}{\rho A△t}$$

where *m* (kg) is the mass quality of infiltration water, *A* (m^2^) is the effective membrane surface area, *ρ* is the density of water (1.0 × 10^3^ kg m^−3^), and Δ*t* (h) is the filtration time. The BSA rejection rate *R* (%) of the membrane was calculated according to Formula (3):

(3) $$R=\left(1-\frac{C_{p}}{C_{f}}\right)\times 100\%$$

where *C_f_* is the BSA concentration in the original solution, mg L^−1^, and *C_p_* is the BSA concentration in the solution after penetration, which was measured in terms of the absorbance at 278 nm using ultraviolet–visible (UV–vis) spectrophotometry.

The antifouling performance of the emulsified oil wastewater of the membrane was expressed by the flux recovery rate (*FRR*; %), as shown in the Formula (4):

(4) $$\mathrm{FRR}=\frac{J_{wc}}{J_{wv}}\times 100\%$$

where *J_wv_* (LMH bar^−1^) is the initial stable pure water flux and *J_wc_* is the recovered water flux after filtering the emulsified oil wastewater.

The reversible fouling ratio (*R_r_*), irreversible fouling ratio (*R_ir_*), and total fouling ratio (*R*_t_) were calculated using Equations (5)–(7): [59]

(5) $$R_{t}=\left(\frac{J_{\mathrm{wv}}-J_{F}}{J_{wv}}\right)\times 100\%$$

(6) $$R_{r}=\left(\frac{J_{\mathrm{wc}}-J_{F}}{J_{wv}}\right)\times 100\%$$

(7) $$R_{ir}=\left(1-\frac{J_{\mathrm{wc}}}{J_{wv}}\right)\times 100\%$$

where *J_F_* is the emulsified oil solution flux.

## 4. Conclusions

Membranes made from various TB/ZTB ratios were prepared using VIPS and utilized to separate emulsified oil wastewater. The relationships between membrane performance and various preparation parameters were investigated. The blended ZTB/TB MF membranes with zwitterionic properties demonstrated excellent hydrophilicity, high rejection rates, and a high antifouling performance when used for emulsified oil wastewater treatment. The hydrophilicity of the MF membrane gradually decreased upon increasing the ZTB content. The cross-sectional structure of the MF membrane exhibited a spongy bicontinuous structure resulting from the delayed phase separation mechanism during VIPS. Additionally, the blended MF membranes exhibited a significantly greater surface porosity and average surface pore diameter compared with the pristine TB MF membrane. Interactions between foulants and the membrane surface could be fine-tuned by regulating the surface charge and hydrophilicity in a synergistic manner, which can prevent irreversible fouling. This study demonstrates the potential for the development of high-performance MF membranes in the treatment of emulsified oily wastewater.

## Figures and Tables

**Table 1:** The surface porosity (*P_s_*), average surface pore size (*r*_s_), maximum surface pore size (*r*_max_), and top-layer thickness (*T*) of the membranes.

Membranes	*P_s_*/%	*r_s_*/μm	*r*_max_/μm	*T*/μm
M0	1.2 ± 0.2	0.178 ± 0.002	0.462 ± 0.008	0.035 ± 0.004
M1	1.6 ± 0.1	0.203 ± 0.001	0.550 ± 0.039	0.030 ± 0.002
M2	2.9 ± 0.1	0.212 ± 0.008	0.660 ± 0.117	0.030 ± 0.005
M3	5.1 ± 0.1	0.247 ± 0.005	0.867 ± 0.070	0.025 ± 0.005
M4	3.9 ± 0.1	0.263 ± 0.006	0.963 ± 0.025	0.055 ± 0.011
M5	5.7 ± 0.4	0.278 ± 0.001	0.992 ± 0.209	0.036 ± 0.007
M6	4.0 ± 0.1	0.293 ± 0.001	1.311 ± 0.103	0.050 ± 0.012
M7	2.7 ± 0.1	0.214 ± 0.001	0.621 ± 0.035	0.021 ± 0.003

**Table 2:** Average surface roughness (*Ra*), root mean square roughness (*Rq*), and interfacial free energy (−*G*_ML_).

Membranes	*Ra* (nm)	*Rq* (nm)	−Δ*G*_ML_ (mJ m^−2^)
M0	21.79 ± 2.22	29.57 ± 2.24	83.0
M1	22.38 ± 2.08	33.50 ± 3.34	95.9
M2	23.73 ± 3.35	34.76 ± 3.90	111.1
M3	24.24 ± 3.06	45.93 ± 1.25	118.1

**Table 3:** Previous literature on the permeate flux and oil rejection of oil–water separation membranes.

Membrane Material/Fabrication	Oil/Surfactant Content	Driving Force (bar)	Separation Efficiency (%)	Flux (LMH bar^−1^)	Flux Recovery Rate (%)	Refs
PVDF (VIPS + TIPS/VIPS + NIPS)	SDS:oil = 1:6 (*w*/*w*)	0.2	/	~3028	~77%	[31]
PSF(VISP)	SDS:oil = 1:99 (*w*/*w*)	0.2	~98.48	~501.89	~49.57%	[52]
PPSU/SPSf (V-LIPS)	Water:oil = 1:99 (*w*/*w*)	0.2	99.5–99.5	508.4~414.1	/	[53]
PVDF-*co*-HFP(VIPS)	oil/water = 1% (*v*/*v*)	1.0	99.5%	600	/	[54]
PVDF/PHEMA (VIPS)	20 mg SDS + 10 mL oil + 990 m water	1.0	99.1% (crude oil)	1866 ± 162 (pump oil)	/	[55]
zwitterionization PVDF(VIPS)	oil/water = 1:99 (*w*/*w*)	0.5	99.0%	180–240		[27]
tannic acid deposited onto PVDF MF membrane	Tween-80 + 2-dichloroethane/hexane/iso-octane and water (*v*/*v*/*v* = 1:50:0.02)	0.8	98%	38 ± 13~401 ± 97	84	[56]
PMCSMA grafted PES MF membrane	Span-80 (4000 mg/L) + Kerosene (50 mg/L)	0.25	99.5%	43	/	[1]
Polydopamine/polyelectrolyte co-deposited onto PP MF membrane	SDS(1000 mg/L) + Oi/waterl (*v:v* = 1:5/)	/	99%	0.65	/	[57]
Zwitterionic Tröger’s base/VISP	SDS(50 mg/L) + Cutting oil(50 mg/L)	0.1	99%	1328	74%	This work

**Table 4:** Compositions and preparation conditions of the casting solution (constant RH = 90%).

Membranes	Composition			Temperature of VIPS Chamber	Exposure Time
	TB (wt%)	ZTB (wt%)	NMP (wt%)	*T*v (°C)	*t*_e_ (min)
M0	18	0	82	50	10
M1	17	1	82	50	10
M2	16	2	82	50	10
M3	15	3	82	50	10
M4	16	2	82	50	5
M5	16	2	82	50	15
M6	16	2	82	30	10
M7	16	2	82	80	10

**Table 5:** Particle size distribution of emulsified oil droplets.

Emulsified Oil Droplet Size (μm)	D10	D50	D90	D(3,2)	D(4,3)
Feed liquid	0.171	0.209	0.266	0.208	0.214

## Data Availability

Data are contained within the article.
